# Behavioural Changes in Mice after Getting Accustomed to the Mirror

**DOI:** 10.1155/2020/4071315

**Published:** 2020-02-03

**Authors:** Hiroshi Ueno, Shunsuke Suemitsu, Shinji Murakami, Naoya Kitamura, Kenta Wani, Yu Takahashi, Yosuke Matsumoto, Motoi Okamoto, Takeshi Ishihara

**Affiliations:** ^1^Department of Medical Technology, Kawasaki University of Medical Welfare, Okayama 701-0193, Japan; ^2^Department of Psychiatry, Kawasaki Medical School, Kurashiki 701-0192, Japan; ^3^Department of Neuropsychiatry, Graduate School of Medicine, Dentistry, and Pharmaceutical Sciences, Okayama University, Okayama 700-8558, Japan; ^4^Department of Medical Technology, Graduate School of Health Sciences, Okayama University, Okayama 700-8558, Japan

## Abstract

Patients with brain function disorders due to stroke or dementia may show inability to recognize themselves in the mirror. Although the cognitive ability to recognize mirror images has been investigated in many animal species, the animal species that can be used for experimentation and the mechanisms involved in recognition remain unclear. We investigated whether mice have the ability to recognize their mirror images. Demonstrating evidence of this in mice would be useful for researching the psychological and biological mechanisms underlying this ability. We examined whether mice preferred mirrors, whether plastic tapes on their heads increased their interest, and whether mice accustomed to mirrors learnt its physical phenomenon. Mice were significantly more interested in live stranger mice than mirrors. Mice with tape on their heads spent more time before mirrors. Becoming accustomed to mirrors did not change their behaviour. Mice accustomed to mirrors had significantly increased interest in photos of themselves over those of strangers and cage-mates. These results indicated that mice visually recognized plastic tape adherent to reflected individuals. Mice accustomed to mirrors were able to discriminate between their images, cage-mates, and stranger mice. However, it is still unknown whether mice recognize that the reflected images are of themselves.

## 1. Introduction

Many scientists have used mirrors to investigate whether animals have visual self-cognitive abilities. Visual self-cognition is the ability to understand the appearance of the self. In general, in an animal, when investigating the presence of the cognitive ability to recognize the mirror image of itself, two methods of verification are used. The first method evaluates whether a self-directed reaction suggesting that the animal recognizes the mirror image as its own reflection is seen, and the second is whether the animal passes the mark test. In the mark test, a mark is made on the body of the target animal, and the animal is put in front of a mirror. The animal is then observed to see whether it inspects the mark or tries to touch it with a part of its body [1]. Using this test, it is possible to judge whether animals understand that the marks reflected in the mirror are attached to their own bodies and not to other individuals. Humans do not pass the mark test until they are two years old [2, 3]. Apart from humans, chimpanzees [4], bonobos [5], orangutans [6], gorillas [7], bottlenose dolphins [8], Asian elephants [9], and Castellus magpies have been shown to pass the mark test [10].

However, some research findings show that mirror self-recognition may not only be the privilege of homeothermic mammals and birds, but that it is also enabled by learnt experiences in some animals. When a mark was placed on the invisible part of squids (*Sepioteuthis lessoniana*), the marked squids stopped in front of the mirror [11]. In recent years, it has also been reported that the cleaner wrasse (*Labroides dimidiatus*) is able to self-recognize by learning [12]. Furthermore, it has been reported that three kinds of ant species also recognized the point drawn on their heads by the reflection of the mirror [13]. There is a possibility that more animals are equipped with mirror self-recognition abilities than previously thought.

The mirror self-recognition test is a visually dependent test; therefore, it yields a false negative result in nonvisually dependent animals [14]. Furthermore, some animals that are not interested in the mark do not change behaviour [14]. Therefore, it has been pointed out that the mirror test is an effective test, but it should not be used as the only indicator of self-awareness [15]. The mark test and mirror test also need to be considered as part of the self-awareness index.

The neurobiological system of mirror self-recognition may be shared among mammals [16]. Mice are rodents and social animals that are widely used for the study of neuroscience. Recently, it has been reported that mice may also experience ownership of their bodies [17]. Furthermore, mice can recognize others using sight [18]. If mice show mirror self-recognition, this will aid in the search for the psychological and biological mechanisms underlying this cognitive ability. Hence, in this study, we investigated whether mice showed mirror self-recognition-like behaviour through a series of experiments.

Since it is difficult for a human observer to objectively interpret the behaviour of mice, this study used indices and analysed the number of times the subject mouse approached the target area, and the time spent there. First, we investigated whether the subject mouse was interested in a mirror over a board. Next, we examined whether the mouse was more interested in a live stranger mouse or a mirror.

Using a method similar to the traditional mark test, we attached a small plastic tape to the head of the mouse and investigated whether the mouse showed an increased interest in the mirror. To clarify whether mice can learn the reflective property of mirrors, we placed a mirror overnight in the home cage of the mouse. We then investigated whether there were changes in the behaviour of the mouse during the tape on the head test. Furthermore, to investigate whether mice can recognize that the reflected images in the mirror are of themselves, we used their own photos and photos of conspecifics (cage-mate and stranger mice) and examined the degree of interest the mice showed to each photo. The purpose of this research was to clarify the possibility of the self-cognitive ability of mice.

## 2. Methods

### 2.1. Animals

All animal experiments were performed in accordance with the U.S. National Institutes of Health (NIH) Guide for the Care and Use of Laboratory Animals (NIH Publication No. 80-23, revised in 1996) and approved by the Committee for Animal Experiments at the Kawasaki Medical School Advanced Research Centre. All efforts were made to minimize the number of animals used and their suffering. One hundred C57BL/6N male mice aged 10 weeks were purchased (Charles River Laboratories, Kanagawa, Japan) and housed in cages (5 animals per cage) with food and water ad libitum under a 12 h light/dark cycle at 23°C to 26°C temperature. Ten mice (*n* = 10) were used for all tests, except for the tape on the head test, where 10 subject mice (*n* = 10) and 10 control mice (*n* = 10) were used. Naive mice were used in all behavioural experiments, which were conducted in behavioural testing rooms between 09.00 and 17.00 h during the light phase of the circadian cycle. After the experiments, all the equipment was cleaned with 70% ethanol and super hypochlorous water to prevent bias based on olfactory cues. Behavioural tests were performed in the order described below.

### 2.2. Rearing with a Mirror Overnight

A mirror (8 × 12 × 0.2 cm) was attached to the side of the mouse breeding cage (18 × 26 × 13 cm) and left this there overnight.

### 2.3. Board and Mirror Preference Test

**Figure 1:**
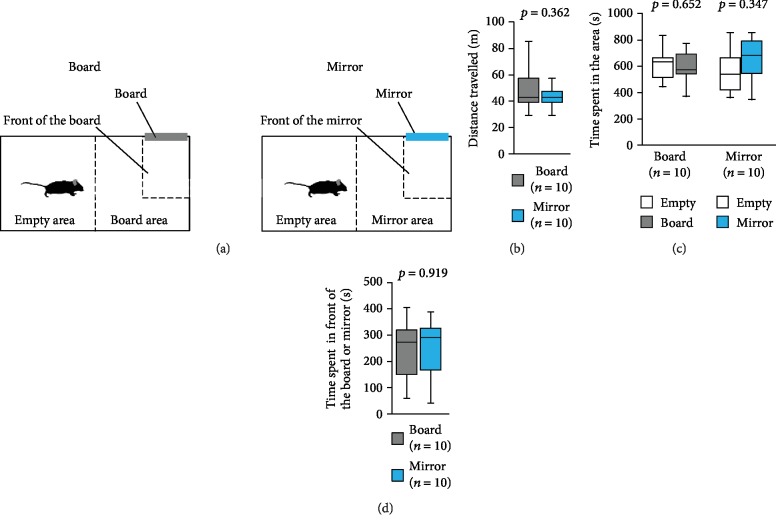
Board preference test and mirror preference test. (a) The schematic diagram of the test. The board area refers to the half of the test apparatus containing the board, and the empty area refers to the other half of the test apparatus. The mirror area refers to the half of the test apparatus containing the mirror, and the empty area refers to the other half of the test apparatus. Graphs showing (b) the total distance travelled, (c) the time spent in the area, and (d) the time spent in front of the board or mirror. All data are presented as box plots. The *p* values were calculated using Student's *t*-test (b and d) and the paired *t*-test (c).

The apparatus consisted of a rectangular box (30 × 60 × 40 cm). An opaque grey plastic board (8 × 12 × 0.2 cm) was affixed to one wall (Figure 1(a)). Each mouse was placed in the box for 5 min and allowed free exploration to habituate. The subject mouse was then placed at the centre of the box and was allowed to explore the entire box for 20 min. One side of the rectangular area was identified as the board area and the other as the empty area. The area 20 cm in front of the board was designated as the board area. The amount of time spent in each area and in front of the board during the 20 min sessions was measured. After the first 20 min session, a mirror was placed instead of the board. The subject mouse was placed at the centre of the box and was allowed to explore the entire box for a further 20 min. The amount of time spent in each area during the second 20 min session was measured as described above. All the components of the apparatus were cleaned after each phase of this test. The data were video recorded and analysed using a video tracking software (ANY-maze, Stoelting Co., Wood Dale, IL, USA).

### 2.4. Going behind the Mirror Test

**Figure 2:**
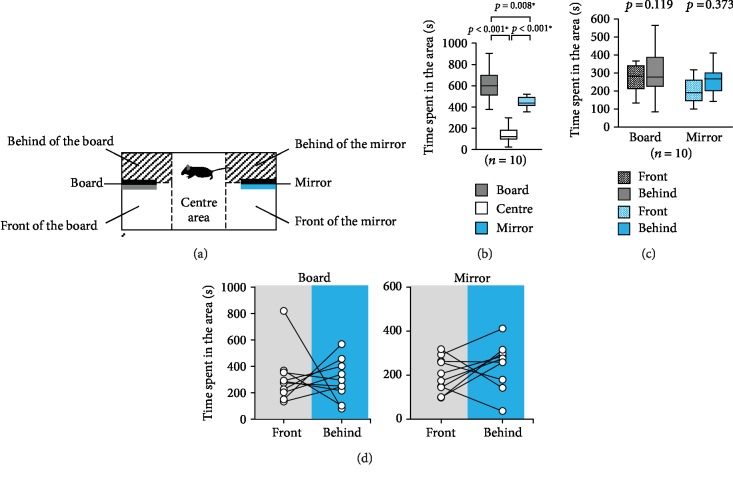
Going behind the board and mirror test. (a) The schematic diagram of the apparatus used in this experiment. An opaque board and a mirror are placed at two sides of a rectangular apparatus, and the apparatus is divided into three parts that represent the board, centre, and mirror areas. Areas in front of and behind the board or mirror were also set. Graphs showing (b) the time spent in the area and (c) the time spent in front of and behind the board or mirror. (d) Graph showing the individual times spent in front of and behind the board or mirror in the going behind the board and mirror test. All data are presented as box plots. ∗ represents a significant difference compared to the controls (*p* < 0.05). The *p* values were calculated using one-way ANOVA (b and d) and two-way ANOVA (c).

The apparatus consisted of a rectangular box (30 × 60 × 40 cm). A mirror (8 × 12 × 0.2 cm) and an opaque board (8 × 12 × 0.2 cm) were placed in each lateral compartment 10 cm from the wall (Figure 2(a)). The subject mouse was placed in the middle chamber and was allowed to explore the entire box for 10 min. The rectangular box was divided into three areas: the board, centre, and mirror areas. We separated the areas further into the front and back of the board and the front and back of the mirror (Figure 2(a)). The amount of time spent in each area during each 10 min session was measured. The data were video recorded and analysed using the ANY-maze software.

### 2.5. Stranger or Mirror Test

**Figure 3:**
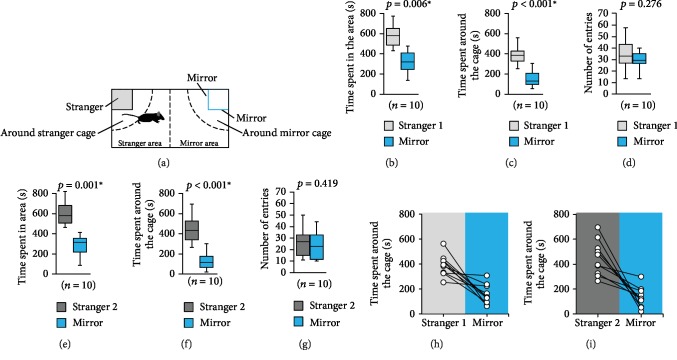
Stranger or mirror test. (a) The schematic diagram of the apparatus used in this experiment. Graphs showing (b) the time spent in the area, (c) the time spent around the cage, and (d) the number of entries around the cage in the first session. Graphs showing (e) the time spent in the area, (f) the time spent around the cage, and (g) the number of entries around the cage in the second session. (h and i) Graphs showing the individual times spent in front of the board or mirror in the stranger or mirror test. All data are presented as box plots (b–g). ∗ represents a significant difference compared to the controls (*p* < 0.05). The *p* values were calculated using the paired *t*-test (b–g).

The apparatus consisted of a rectangular box (30 × 60 × 40 cm). Two transparent cages (7.5 × 7.5 × 10 cm with several holes of 1 cm diameter each) were placed at both ends of the rectangular apparatus (Figure 3(a)). Each subject mouse was placed in the box for 10 min and allowed free exploration to habituate. In the first session, a stranger mouse (stranger 1) was put into one of the cages and two mirrors were put around the other cage (Figure 3(a)). The stranger mouse was enclosed in the transparent cage, which allowed the subject and stranger mice to have nose contact between the bars but prevented fighting. The subject mouse was placed in the centre and allowed to explore the entire box for 10 min. In the second session, stranger 1 mouse was replaced by another stranger mouse (stranger 2). One side of the rectangular area was identified as the stranger area and the other as the mirror area. The amount of time spent in each area and around each cage during the 10 min sessions was measured. The apparatus was cleaned after each phase of the test. The data were recorded on video and analysed using the ANY-maze software.

### 2.6. Tape on the Head Test

**Figure 4:**
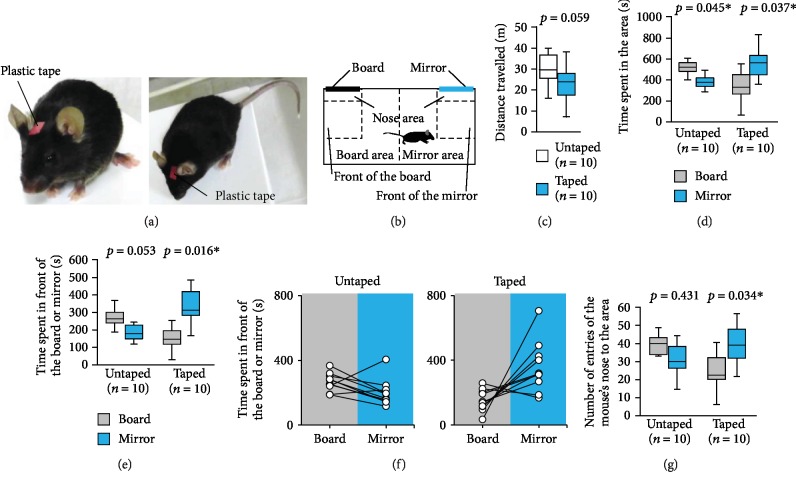
Tape on the head test. (a) A sample picture of a mouse marked with a plastic tape. (b) The schematic diagram of the apparatus used in this experiment. Graphs showing (c) the total distance travelled, (d) the time spent in the area, (e) the time spent in front of the board or mirror, and (g) the number of times the mouse's nose entered the area. (f) Graph showing the individual times spent in front of the board or mirror by the control and taped mice in the tape on the head test. All data are presented as box plots (c–e, g). ∗ represents a significant difference compared to the controls (*p* < 0.05). The *p* values were calculated using Student's *t*-test (c) and the paired *t*-test (d, e, and g).

Using a method similar to the traditional mark test [4], we stuck a piece of tape to the head of a mouse and analysed its behaviour. The apparatus consisted of a rectangular box (30 × 60 × 40 cm). A mirror (8 × 12 × 0.2 cm) and an opaque board (8 × 12 × 0.2 cm) were attached to the wall of each lateral compartment (Figure 4(b)). Each mouse was placed in the box for 6 min and allowed free exploration to habituate. Rectangular red plastic tapes (2 × 5 mm; No. 360; Sekisui Chemical Co., Tokyo. Japan) that would adhere to the mice were prepared and stuck on the head of the subject mice (Figure 4(a)). We did not stick tape to the control mice but touched their heads. During the test session, the mouse was placed in the middle area and allowed to explore the entire box for 10 min. The amount of time spent in each area and in front of the board or the mirror during the 10 min session was measured. The number of times that the mouse's nose entered the area (within 5 cm from the mirror or board; Figure 4(b)) were measured. The data were recorded on video and analysed using the ANY-maze software.

### 2.7. My-Photo Recognition Test

**Figure 5:**
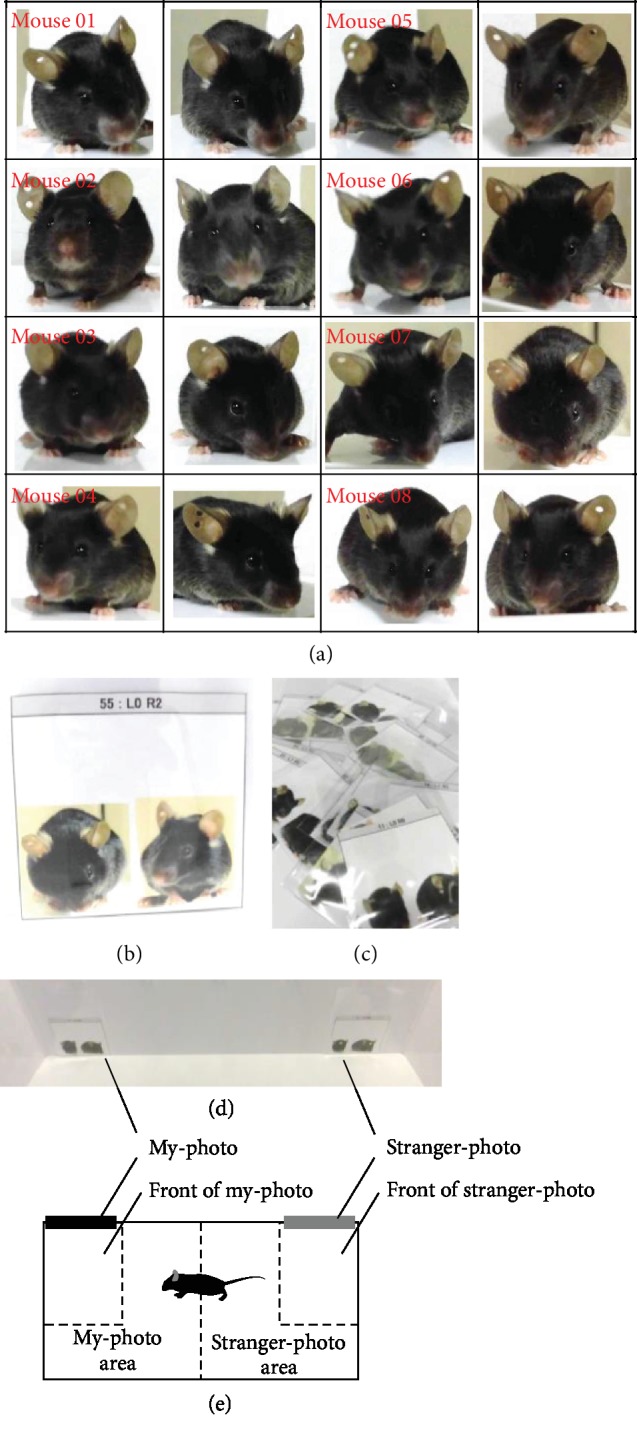
Sample photo and schematic diagram of the photo discrimination test. (a) A sample picture of subject mice (1-8). (b) A sample life-size photo from the front of the mouse used in the experiment. (c) Photos of each subject mouse. (d) The photo of the subject mouse (my-photo) and that of a stranger mouse (stranger-photo) are placed on both sides of the experimental apparatus. (d) The schematic diagram of the apparatus used in this experiment.

We took a photograph of the front of the mouse and made a life-sized print-out of this (Figures 5(a)–5(c)). Pictures of the mouse were affixed to both ends of the rectangular experimental apparatus (Figures 5(d) and 5(e)). We attached pictures of the subject mouse (my-photo) and a stranger mouse (stranger-photo) for the my-photo versus stranger-photo test. The subject mouse was placed in the centre and allowed to explore the entire box for 10 min. One side of the rectangular area was identified as the my-photo area and the other as the stranger-photo area. The amount of time spent in each area and in front of each photo during the 10 min session was measured. In the my-photo versus cage-mate photo test, we attached pictures of the subject mouse (my-photo) and a cage-mate mouse (cage-mate photo) to both ends of the apparatus. The subject mouse was placed in the centre and allowed to explore the entire box for 10 min. One side of the rectangular area was identified as the my-photo area and the other as the cage-mate photo area. The amount of time spent in each area and in front of each photo during the 10 min session was measured. The apparatuses were cleaned after each phase of the test. The data were recorded on video and analysed using the ANY-maze software.

### 2.8. Statistical Analysis of the Behavioural Tests

Statistical analysis was conducted using the SPSS software (IBM Corp., Tokyo, Japan). Data were analysed with the repeated measures analysis of variance (ANOVA) and two-way ANOVA, followed by Fisher's LSD test, Student's *t*-test, or paired *t*-test. A *p* value of less than 0.05 was regarded as statistically significant. Data are shown as box plots.

## 3. Results

### 3.1. Mirror Preference Test

First, we tested whether mice were interested in the mirror by placing an opaque board or a mirror and examining the time spent in the front of each of these (Figure 1(a)). We observed no significant difference between the total distances travelled when the board was placed and when the mirror was placed (Figure 1(b), *t*_9_ = 0.961, *p* = 0.362). When the board was placed, mice spent a similar amount of time in the empty area as in the board area (Figure 1(c), *t*_9_ = 0.467, *p* = 0.652). Likewise, when the mirror was placed, mice spent a similar amount of time in both areas (Figure 1(c), *t*_9_ = −0.992, *p* = 0.347). There were no significant differences between the times spent in front of the board and in front of the mirror (Figure 1(d), *t*_9_ = −0.105, *p* = 0.919).

### 3.2. Going behind the Mirror Test

In this experiment, we investigated whether mice frequently went behind the mirror, if space was present behind it. There were no significant differences between the times spent in the board, centre, and mirror areas (Figure 2(b), *F*_2,27_ = 33.84, *p* < 0.001; board vs. centre: *p* < 0.001; board vs. mirror: *p* = 0.008; centre vs. mirror: *p* < 0.001). There were no significant differences between the times spent in front of and behind the board (Figures 2(c) and 2(d), *F*_1,36_ = 2.546, *p* = 0.119) and between the times spent in front of and behind the mirror (Figures 2(c) and 2(d), *F*_1,36_ = 0.811, *p* = 0.373).

### 3.3. Stranger or Mirror Test

We investigated whether the interest that the subject mouse showed towards a mirror was equivalent to the interest it showed towards a live stranger mouse. In the first session, subject mice spent a significantly longer amount of time in the stranger area containing the transparent cage with the live mouse (stranger 1) than in the mirror area containing the cage with the mirror (Figure 3(b), *t*_9_ = 3.537, *p* = 0.006). Moreover, subject mice spent significantly more time around the cage containing the stranger 1 mouse than around the cage containing the mirror (Figures 3(c) and 3(h), *t*_9_ = 4.996, *p* < 0.001). The number of entries of the subject mice around the cage containing the stranger 1 mouse was greater than that around the mirror cage (Figure 3(d), *t*_9_ = 1.161, *p* = 0.276).

In the second session, subject mice spent a significantly longer time in the stranger area containing the transparent cage with the live mouse (stranger 2) than in the mirror area containing the mirror cage (Figure 3(e), *t*_9_ = 4.551, *p* = 0.001). Subject mice also spent significantly more time around the cage containing the stranger 2 mouse than around the mirror cage (Figures 3(f) and 3(i), *t*_9_ = 4.924, *p* < 0.001). There was no significant difference in the number of entries around the cage containing the stranger 2 mouse and that around the mirror cage (Figure 3(g), *t*_9_ = 0.847, *p* = 0.419).

### 3.4. Tape on the Head Test

Next, we investigated whether mice increased their interest in the mirror after having a plastic tape attached to their heads (Figure 4(a)), and by affixing a board and mirror to two sides of the experimental apparatus (Figure 4(b)).

We observed no significant difference between the total distance travelled by the taped mice and the untaped mice (Figure 4(c), *t*_9_ = 2.009, *p* = 0.059). Untaped mice spent a significantly longer time in the area containing the board than in the area containing the mirror (Figure 4(d), *t*_9_ = 2.327, *p* = 0.045). Contrarily, taped mice spent a significantly longer time in the area containing the mirror than in the area containing the board (Figure 4(d), *t*_9_ = −2.443, *p* = 0.037). Untaped mice spent a similar amount of time in front of and behind the mirror and in front of and behind the board (Figures 4(e) and 4(f), *t*_9_ = 2.227, *p* = 0.0529). Taped mice spent significantly more time in the front of the mirror than in front of the board (Figures 4(e) and 4(f), *t*_9_ = −2.961, *p* = 0.016). No differences were observed in the number times the untaped mouse's nose entered the area (Figure 4(g), *t*_9_ = 0.983, *p* = 0.431). Taped mice approached the front of the mirror with their noses more frequently (Figure 4(g), *t*_9_ = −2.247, *p* = 0.034). The taped mice did not show behaviour suggestive of trying to eliminate the tape. As in the traditional mark test, the taped mice did not show self-directed behaviour (paying attention to the mark on their body, e.g., touching mark and observing mark).

### 3.5. Tape on the Head Test after Spending the Night with the Mirror

**Figure 6:**
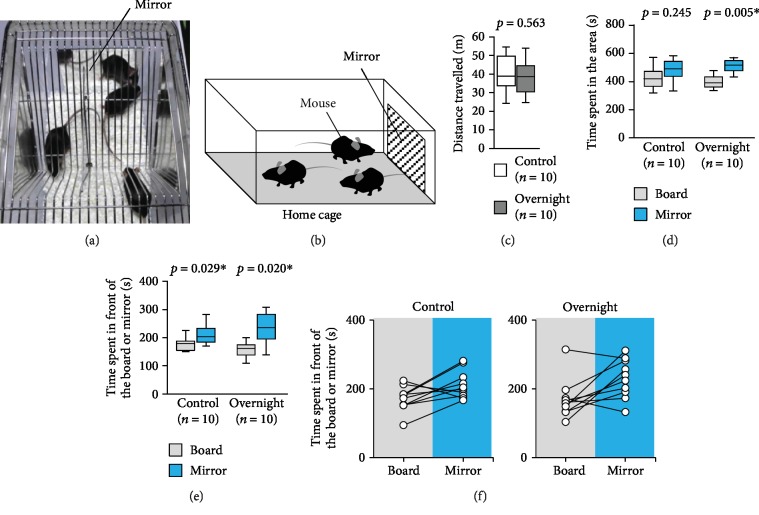
Tape on the head test after they spent the night with the mirror. (a) A sample picture of a home cage with a mirror. (b) The schematic diagram of the home cage with the mirror. Graphs showing (c) the total distance travelled, (d) the time spent in the area, and (e) the time spent in front of the board or mirror. (g) Graph showing the individual times spent in front of the board or mirror during the tape on the head test by control taped mice that did not spend the night with the mirror and overnight taped mice that spent the night with the mirror. All data are presented as box plots (c–e). ∗ represents a significant difference compared to the controls (*p* < 0.05). The *p* values were calculated using Student's *t*-test (c) and the paired *t*-test (d and e).

We performed the tape on the head test after the mice were fully familiarized with the mirror. We attached a mirror to the breeding cage and left this there overnight (Figures 6(a) and 6(b)). We performed the test on mice that were not kept with mirrors (control taped mice) and mice kept with mirrors (overnight taped mice).

We observed no significant difference in the total distances travelled between the two groups (Figure 6(c), *t*_9_ = 0.589, *p* = 0.563). The control taped mice spent a similar amount of time in the area containing the board as in the area containing the mirror (Figure 6(d), *t*_9_ = −1.244, *p* = 0.245). The control taped mice spent a significantly longer time in front of the mirror than in front of the board (Figures 6(e) and 6(f), *t*_9_ = −2.589, *p* = 0.029). The overnight taped mice spent a significantly longer time in the area containing the mirror than in the area containing the board (Figure 6(d), *t*_9_ = −3.744, *p* = 0.005). They spent significantly more time in front of the mirror than in front of the board (Figures 6(e)and 6(f), *t*_9_ = −2.813, *p* = 0.020). Both groups of mice did not show behaviour suggestive of trying to eliminate the tape.

### 3.6. My-Photo Recognition Test with My-Photo and Stranger-Photo

In this experiment, we investigated whether mice accustomed to the mirror could distinguish between the my-photos and the stranger-photos. Mice can distinguish pictures by two-dimensional visual stimulation [19]. Furthermore, recent studies have shown that mice can recognize virtual reality spaces [20, 21]. We attached pictures of the subject mouse (my-photo) and a stranger mouse (stranger-photo) on the wall of the rectangular experimental apparatus (Figures 5(d) and 5(e)). We tested with mice bred without mirrors (mice unaccustomed to mirrors) and mice bred with mirrors (mice accustomed to mirrors).

**Figure 7:**
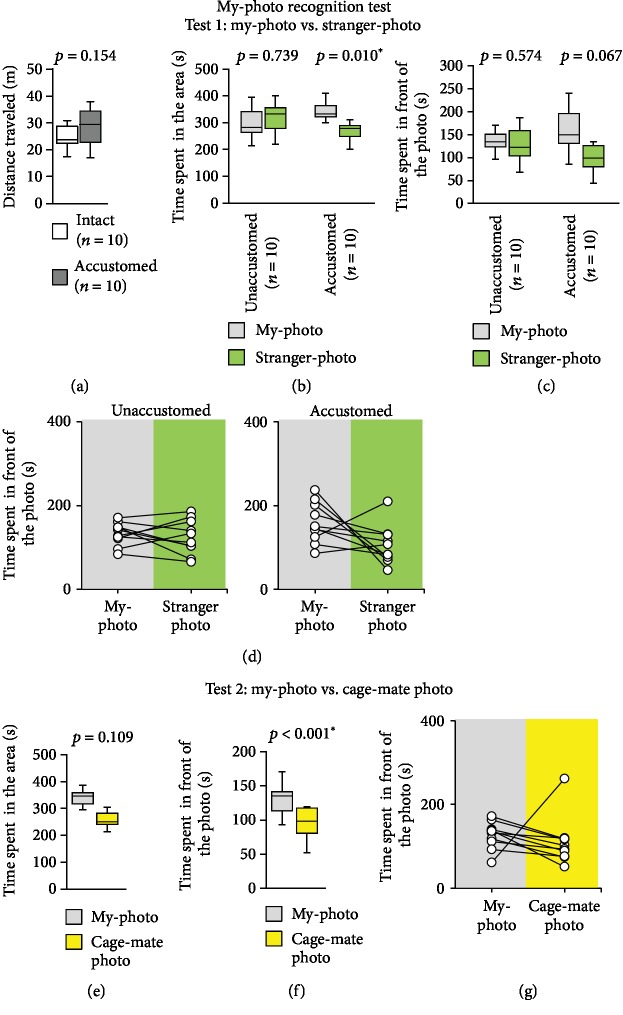
My-photo discrimination test. My-photo versus stranger-photo test: graphs showing (a) the total distance travelled, (b) the time spent in the area, and (c) the time spent in front of the photo. (d) Graph showing the individual times spent in front of the photo by both control mice and mice that spent the night with a mirror in this test. My-photo versus cage-mate photo test: graphs showing (e) the time spent in the area and (f) the time spent in front of the photo. (g) Graph showing the individual times spent in front of the photo by both control mice and mice that spent the night with a mirror in this test. All data are presented as box plots (a–c, e, and f). ∗ represents a significant difference compared to the controls (*p* < 0.05). The *p* values were calculated using Student's *t*-test (a) and the paired *t*-test (b, c, e, and f).

We observed no significant difference in the total distances travelled between the two groups (Figure 7(a), *t*_18_ = −1.487, *p* = 0.154). The mice unaccustomed to mirrors spent a similar amount of time in the area containing the my-photo and in the area containing the stranger-photo (Figure 7(b), *t*_9_ = −0.344, *p* = 0.739). They spent a similar amount of time in front of the my-photo and in front of the stranger-photo (Figures 7(c) and 7(d), *t*_9_ = 0.584, *p* = 0.574). The mice accustomed to mirrors spent a significantly longer time in the area containing the my-photo than in the area containing the stranger-photo (Figure 7(b), *t*_9_ = 3.231, *p* = 0.010). They spent a similar amount of time in front of the my-photo and in front of the stranger-photo (Figures 7(c) and 7(d), *t*_9_ = 2.079, *p* = 0.067).

### 3.7. My-Photo Recognition Test with My-Photo and Cage-Mate Photo

Next, we investigated whether mice accustomed to the mirror could distinguish between my-photos and cage-mate photos. We attached pictures of the subject mouse (my-photo) and a cage-mate mouse (cage-mate photo) on the wall of the rectangular experimental apparatus. We tested with mice bred with mirrors (mice accustomed to mirror). Mice accustomed to mirrors spent a similar amount of time in the area containing the my-photo and in the area containing the cage-mate photo (Figure 7(e), *t*_9_ = 1.777, *p* = 0.109). However, mice accustomed to mirrors spent significantly more time in front of the my-photo than in front of the cage-mate photo (Figures 7(f) and 7(g), *t*_9_ = 5.232, *p* < 0.001).

## 4. Discussion

In this study, we applied a plastic tape to the heads of mice and investigated their change in interest towards the mirror. The interest in the mirror when the plastic tape was applied to the heads of mice significantly increased from before they were accustomed to the mirror to after they were accustomed to the mirror. Furthermore, we found that mice frequently contacted the mirror, suggesting that they could distinguish the image on the mirror from the faces of the cage-mate and stranger mice.

Animals that are thought to be able to perceive their reflections in the mirror as their own figures, in many cases, follow four steps when faced with a mirror: (1) make social reactions, (2) explore the physical sense (such as checking the back of the mirror), (3) perform repetitive actions to test the mirror, and (4) understand that the image reflected is their own [9]. In the tests used in this study, mice did not show social reactions or exploratory behaviours of reacting positively to mirrors as did chimpanzees, dogs, and fish in previous studies. The interest of the mice to the opaque board was comparable to that to the mirror. Previous reports show that the mirror slightly disgusted the mice, and that unlike with other animal species, mirrors are not environmentally enriching material for mice [22, 23]. For this reason, chambers composed of mirrors are used to test the effects of anxiolytic drugs in mice [24, 25]. The difference in our results may be due to the differences in the familiarity of the mice to the mirror, the reflective state of the plastic breeding home cages, single breeding versus mass breeding, and the illuminance of the experimental environment. Specular reflection provides only visual information, whereas live animals provide multiple sensory information. Therefore, live animals have richer stimuli than mirrors. Since mice are animals that prioritize olfaction rather than vision and hearing, it is considered reasonable that their interest in the mirror without smell quickly diminishes.

In this study, all the mice showed a stronger interest in the live stranger mouse than in the mirror. Previous studies have reported that mice are more interested in mirrors than stranger mice [26]. The difference in these results may have been because of accustoming the mice to the mirror, which may have affected the result. However, it is reasonable that the mice would show interest in live stranger mice that provide multiple sensory information rather than in specular reflections that provide only visual information. Moreover, even if mice do not understand the reflection in the mirror as the reflected image of itself, it is a natural reaction to ignore the mirror image as a harmless stimulus for themselves rather than to recognize it as a homogeneous individual to react with socially [27]. In fact, the mental process and cognitive ability of the mouse in response to the mirror are unknown, and further research is needed for elucidation. This study shows that mice are not interested in mirrors.

We applied small plastic tapes to the heads of mice for the tape on the head test. Mice with the tapes on their heads showed an increased interest in the mirror. Even after becoming accustomed to the mirror, the interest of the taped mouse to the mirrors remained high. However, the taped mice did not show behaviour suggestive of trying to eliminate the tape. During the mark test, the mark may not be perceived as abnormal by the animals, and they may not feel the impulse to touch it. Pigs have been reported to be able to recognize their movements in the mirror in a very short time, and to be self-conscious [28]. However, it is thought that since pigs are accustomed to the state of mud being attached to their bodies, even if they are marked during experiments, they do not mind this. This does not mean that they are not self-perceiving. Even if they can perceive themselves, if the motivation to remove dirt from their faces is small, they would not show action suggestive of trying to touch the mark. It may be possible that mice, like pigs, do not feel the necessity to remove foreign objects attached to their bodies. The mark test is a compound task in which the ability of the subject to use tools, recognize itself, and detect visual information is questioned. The mark test has been used as a method for confirming the presence or absence of self-awareness, but opinion is divided on its validity [16]. The mark test represents one aspect of self-recognition that has, in recent years, been considered to be different from the overall self-recognition that human beings experience. Moreover, it may not be so meaningful to target an animal that mainly uses a sense other than vision during the mirror test [29]. The results of the present study did not necessarily indicate the existence of self-recognition capability in mice. However, it showed that mice visually perceived unusual states through the mirror. Further research is needed to clarify factors that increase the interest of mice towards mirrors.

Other than the head, the throat is another location that can be used to attach the tape. A similar study analysed the behaviour of a magpie with a tape attached to the throat [10]. However, the throat is a motile part of the animal body, and a tape attached to the throat would provide a tactile stimulus. However, the skin on the head is immobile, and a level of similar tactile stimulus would be implausible.

This study showed that, by spending time with a mirror in the home cage, mice changed their interest in photos of themselves over photos of cage-mates and stranger mice. This mouse behaviour indicated that by learning through the mirror, the mice recognized that the image in the mirror was different from the figure of their cage-mate mice. It should be noted that the results of this study are not evidence that mice recognize the images in the mirror as their own. However, some animals have shown that their self-perception of the mirror image can be enabled through experience [30, 31]. It has been reported that self-perception of the mirror image occurs naturally in rhesus macaques after training for accurate visual-specific receptor association to mirror images [32, 33]. It has also been reported that pigeons pass the mark test after thorough training by voluntary and mirror-based pecking [30, 34]. In recent years, it has been reported that the cleaner wrasse (*Labroides dimidiatus*) is able to self-recognize by learning [12]. In addition, it is indicated that the age of acquiring mirror image self-recognition in humans is related to the frequency of postnatally experiencing the mirror and to cultural differences [35]. Among infants in Africa, where opportunities to see mirrors are less frequent than in developed countries, it is reported that the age at which mirror images of the self can be clearly perceived is somewhat higher. The results of this study are consistent with previous reports, indicating the possibility that more animals show that there is a sense of “self” than we think. Since mirror image self-recognition increased as the mirror was experienced more frequently, it is considered that changing the cognitive evaluation of the mirror at the stage when the reflecting property of the mirror and the reflecting object are learnt becomes the turning point.

The mental processes of mice and other animal species, such as apes are unknown, and it is difficult to decipher the cognitive abilities of such animals. Our results show that the mouse is an animal that alters recognition to the mirror by learning. Further research is needed to clarify the mirror recognition of the self by mice. Having a mouse as an effective model for behavioural research, such as mirror self-recognition, opens doors to study aspects of this behaviour that would otherwise be impossible to study.

Patients who suffer from failure of brain function due to a stroke or dementia may show symptoms of being able to recognize the images of their family members and others in the mirror, while not being able to recognize their own images. This phenomenon is called mirror self-misidentification [36]. Mirror self-misidentification is also a symptom of dissociative disorder [37]. However, the mechanism by which this occurs is not clear. Furthermore, when the function of the upper part of the medial prefrontal cortex is temporarily stopped by the transcranial magnetic stimulation method, the person manifests symptoms of being unable to recognize themselves when looking at the mirror [38]. These reports suggest that specific neural circuits are involved in the perception of mirror images of oneself in humans. It is therefore useful to develop a method to clarify these neural mechanisms, to treat cranial nerve disease, and to further clarify the evolutionary basis of the cognitive ability of recognizing mirror images of oneself. New knowledge obtained by conducting experiments on animals focus on whether or not mirror self-recognition is possible for those specific species. Furthermore, many other questions on the neural infrastructure remain. This study shows the potential of using mice for elucidating neural circuits.

## 5. Conclusion

Our results indicated that mice visually recognized plastic tape adhered onto them in their reflection. By getting accustomed to the mirror, mice were able to distinguish between conspecific mice and their reflection in the mirror. However, it is not clear from this study whether mice perceive mirror images of themselves as their own images. Further alternative research is needed to clarify whether mice recognize themselves. Nevertheless, this study showed that mice may be useful as experimental models for elucidating the neural mechanisms of mirror image cognition.

## Data Availability

The datasets generated during and/or analysed during the current study are available from the corresponding author on reasonable request.
